# Synergistic inhibition of agr quorum sensing in methicillin-resistant *Staphylococcus aureus*: a novel approach with natural compound encapsulation

**DOI:** 10.1128/spectrum.01759-25

**Published:** 2025-12-05

**Authors:** Kourosh Naderi, Mohammad Sadegh Damavandi, Atefe Rezaei, Seyd Asghar Havaei

**Affiliations:** 1 Department of Microbiology, School of Medicine, Isfahan University of Medical Sciences 48455, Isfahan, Iran; 2 Antimicrobial Resistance Research Center, Mashhad University of Medical Sciences 113380 , Mashhad, Iran; 3 Department of Microbiology and Virology, Faculty of Medicine, Shahid Hasheminejad Hospital, Mashhad University of Medical Sciences 37552, Mashhad, Iran; 4 Department of Food Science and Technology, School of Nutrition and Food Science, Isfahan University of Medical Sciences 48455, Isfahan, Iran; Guizhou Medical University, Guiyang, China

**Keywords:** antibiotic resistance, MRSA, quorum sensing, biofilms, chitosan

## Abstract

Methicillin-resistant *Staphylococcus aureus* (MRSA) poses a serious threat due to its resistance to common antibiotics. This study investigates the antibacterial and quorum-sensing (QS) inhibitory effects of encapsulated quercetin (QE) and ferulic acid (FA) in chitosan (CS) nanoparticles against MRSA virulence genes. QE and FA were encapsulated into CS nanoparticles and characterized using scanning electron microscope, X-ray diffraction, dynamic light scattering (DLS), thermogravimetric analysis (TGA), and zeta potential analysis. Encapsulation efficiency, antibacterial activity, biofilm inhibition, hemolysis, macrophage intracellular killing, cytotoxicity, and apoptosis induction were assessed for both free and encapsulated forms. Antibacterial activity was evaluated via growth inhibition, biofilm formation assays, and minimum inhibitory concentration (MIC) determination in combination with clindamycin. RT-PCR was used to assess effects on MRSA virulence gene expression. All treatments inhibited MRSA growth, with the CS-QE-FA combination showing the highest inhibition (85%) and biofilm reduction (90%). This combination also synergized with clindamycin, reducing its MIC to 1/64 of the original. RT-PCR revealed significant downregulation of QS-related genes, including *RNAIII*, *agrA*, *hla*, and *psmα* (*P* < 0.001). CS nanoparticles encapsulating QE and FA significantly inhibit MRSA growth and QS-related gene expression, especially in combination. Their synergistic action with clindamycin suggests potential for novel therapeutic strategies against antibiotic-resistant infections.

**IMPORTANCE:**

Antibiotic-resistant infections like those caused by methicillin-resistant *Staphylococcus aureus* (MRSA) are a growing global health concern, often leading to longer hospital stays, increased costs, and higher mortality. This study presents a novel approach using natural compounds—quercetin and ferulic acid—encapsulated in chitosan nanoparticles to target MRSA. Not only do these nanoparticles inhibit bacterial growth and biofilm formation, but they also disrupt the bacteria’s communication system (quorum sensing), which controls the production of toxins. Importantly, these effects are enhanced when combined with clindamycin, a commonly used antibiotic. This combination reduces the amount of antibiotic needed and may help overcome drug resistance. The findings offer a promising strategy for developing safer, more effective treatments against persistent infections while potentially slowing the spread of antibiotic resistance.

## INTRODUCTION

The rapidly increasing threat of antibiotic resistance has reached critical levels and has now become a serious challenge, as we are less capable of dealing with bacterial infections (1–3). Although there are numerous bacterial pathogens, it is methicillin-resistant *Staphylococcus aureus* (MRSA) that captures the attention of scientists because it is resistant to several antibiotics, and consequently, conventional treatment methods are gradually becoming inefficient (4, 5). In the task of developing novel therapy ideas, aiming at the bacterial communication system has been a breakthrough strategy to reduce virulence and make the strain more sensitive to antibiotics (6). Most notably, the interruption of quorum sensing (QS), which is the main mechanism for the communication of bacteria and the expression of virulence factors, has been the focus of many researchers as one of the possible ways to deal with antibiotic-resistant pathogens (7, 8).

*Staphylococcus aureus* (*S. aureus*) ingeniously utilizes a sophisticated quorum-sensing system, including the accessory gene regulator (agr) system, to control the expression of virulence factors, which are important for its pathogenicity and antibiotic resistance (9). The agr system regulates the bacterial communication through both the production and detection of autoinducing peptides (AIPs). When the level of AIPs reaches a certain point, the bacteria respond by initiating signaling pathways, which lead to the expression of virulence factors (10). In addition to regulating virulence, the agr system plays a crucial role in biofilm formation and dispersal. Activation of agr modulates surface proteins and enzymes that influence biofilm architecture, allowing *S. aureus* to form structured, multicellular communities that protect the bacteria from host immune defenses and increase resistance to antibiotics. Biofilms, therefore, contribute significantly to chronic and recurrent MRSA infections, and targeting agr can weaken biofilm integrity while enhancing the efficacy of antibiotic therapy (11, 12).

Natural compounds, with their varied chemical structures and potential for multi-targeted activities, have been regarded as promising molecules to impede bacterial QS (13). Quercetin (QE), one of the well-known flavonoids in common fruits and vegetables, has attracted the interest of scientists since it is capable of anti-QS activity by interfering with AIP-mediated signaling and inhibiting the expression of agr-regulated virulence genes in diverse bacterial pathogens (14).

Similarly, ferulic acid (FA), a common phenolic compound in plant cell walls, has been shown to disrupt QS systems in several bacterial species (15). Chitosan (CS) is also considered a successful analog due to its ability to interfere with bacterial communication. Additionally, CS possesses a positive charge, acting as a natural antimicrobial material and being investigated for drug delivery applications (16, 17). However, the potential synergistic effects of these compounds in controlling MRSA infections, particularly through QS inhibition, remain largely unexplored. The present study, therefore, aims to investigate the potential of QE, FA, and CS, both individually and in combination with clindamycin, as QS inhibitors against MRSA. Specifically, we evaluate their effects on agr-regulated virulence gene expression, biofilm formation, and overall bacterial pathogenicity. Furthermore, by incorporating these compounds into nanoparticles, we aim to enhance their stability, bioavailability, and targeted delivery, ultimately improving their therapeutic efficacy against MRSA infections.

## MATERIALS AND METHODS

### Bacterial strains and culture conditions

MRSA strains were obtained from the American Type Culture Collection (strain number 33592) and clinical isolates. The MRSA strains were maintained on Mueller-Hinton agar plates at 4°C. For experimental use, overnight cultures were prepared by inoculating a single colony from the agar plate into Mueller-Hinton broth. The inoculated broth was incubated at 37°C with agitation at 200 rpm for 16–18 h to achieve exponential phase growth.

### Compounds and encapsulation

QE and FA were obtained from commercial sources (purity ≥98%) and dissolved in dimethyl sulfoxide (DMSO) to prepare stock solutions at concentrations of 10 mg/mL. The solutions were stored at 4°C until use. CS (medium molecular weight, degree of deacetylation 75%–85%) was sourced from Sigma-Aldrich. CS was dissolved in 1% (vol/vol) acetic acid solution to achieve a concentration of 2% (wt/vol). The solution was stirred at room temperature for 4 h until completely dissolved, then filtered through a 0.45 µm membrane to remove any undissolved particles.

CS-encapsulated compounds were prepared by the ionic gelation method, with CS and sodium tripolyphosphate solutions mixed at a ratio of 4:1 (vol/vol) (18, 19). Equal volumes of QE and FA stock solutions were mixed to achieve a final concentration of 5 mg/mL for each compound. This mixed solution was added dropwise to the CS solution under constant stirring (1,000 rpm) at room temperature. The mixture was stirred for an additional 2 h to ensure proper encapsulation of the compounds and formation of nanoparticles. The resulting nanoparticles were collected by centrifugation at 10,000 rpm for 30 min at 4°C. The supernatant was discarded, and the nanoparticles were washed twice with deionized water to remove any unencapsulated compounds. The final nanoparticle suspension was then stored at 4°C until further use. The created complexes include FA combined with CS (FACS), QE combined with CS (QECS), and a combination of CS, FA, and QE (QEFACS).

### Characterization

#### Particle size

Particle size distribution of the nanoparticles was determined using a Mastersizer 2000 (Malvern Instruments Ltd., UK) based on static light scattering, with a dispersant refractive index of 1.33 for water. Each measurement was performed in triplicate, and results were reported as volume mean diameters (D4,3). The zeta potential of the nanoparticles was measured at room temperature (25°C) using a Zetasizer (DLS, Bruker, Germany) with a scattering angle of 90°.

#### Encapsulation efficiency (EE)

The EE of QE and FA was determined by measuring the amount of free (non-encapsulated) compounds in the supernatant after centrifugation. The concentration of free QE and FA was quantified using high-performance liquid chromatography. Encapsulation efficiency was calculated using the following formula:


$$EE\;(\%)=\frac{\left(Total\;compound-Free\;compound\right)}{Total\;compound}\times 100$$

Data were expressed as mean ± standard deviation (SD) from three independent experiments.

#### Scanning electron microscope (SEM) analysis

The surface morphology of CS, QECS, FACS, and QEFACS nanoparticles was examined using a SEM (ZEISS, Germany). Samples were prepared by placing a drop of nanoparticle suspension onto a clean silicon wafer. The wafer was then left to dry at room temperature to ensure even distribution of nanoparticles on the surface. To enhance image quality and reduce charging effects during SEM analysis, the dried samples were coated with a thin layer of gold. This coating was achieved using a Hitachi S-4160 sputter coater (Japan), operating at a voltage of 10 kV for 60 seconds. The coating thickness was carefully controlled to ensure minimal interference with surface details. The SEM images were acquired at various magnifications to assess nanoparticle size, shape, and surface features.

#### X-ray diffraction (XRD) assay

XRD analysis was performed on the FA, QE, CS, QECS, FACS, and QEFACS nanoparticles using a D8 ADVANCE X-ray diffractometer (Bruker, Germany). The analysis utilized Cu-Kα radiation (λ = 1.54 Å) with an operating voltage of 40 kV and a current of 30 mA. Scanning was performed across a 2θ range from 5° to 60°, with a step increment of 0.05°/s.

#### TGA analysis

A thermogravimetric analyzer (TGA-LABSYS EVO, SETARAM, France) was used to examine the thermal properties of the FA, QE, CS, QECS, FACS, and QEFACS nanoparticles. The TGA measurements were carried out over a temperature range of 25 to 600°C, with a steady heating rate of 10°C/min under an argon atmosphere. Weight loss as a function of temperature was recorded to evaluate the thermal decomposition profile.

### The agr QS inhibition assay with RT-PCR

The inhibitory effects of QE, FA, and CS encapsulation on agr QS were evaluated using an MRSA strain. The MRSA strain was grown overnight in Luria-Bertani (LB) broth at 37°C with shaking at 200 rpm. The overnight culture of the MRSA strain was diluted 1:100 in fresh LB broth. Aliquots (1 mL) of the diluted culture were dispensed into sterile 15 mL conical tubes. Test compounds were added to the cultures at final concentrations of 25 µg/mL, 50 µg/mL, and 100 µg/mL for QE and FA. For CS-encapsulated compounds, equivalent concentrations of QE and FA within the CS matrix were used. Control cultures received an equivalent volume of the respective solvents (DMSO or ethanol) without the test compounds. Treated cultures were incubated at 37°C with agitation at 200 rpm for 16 h. After incubation, 100 µL aliquots of each culture were transferred to a 96-well microplate. Bacterial growth was assessed by measuring optical density at 600 nm (OD_600_) using GENESYS 30 spectrophotometer (Thermo Fisher Scientific, Waltham, MA, USA). The percentage inhibition of agr gene expression was calculated relative to the control cultures.

Total RNA was extracted from 1 mL aliquots of treated and control cultures using the RNeasy Mini Kit (Qiagen, Valencia, CA, USA) following the manufacturer’s instructions. RNA concentration and purity were assessed using a NanoDrop spectrophotometer (Thermo Fisher Scientific). cDNA was synthesized from 1 µg of total RNA using the iScript cDNA Synthesis Kit (Bio-Rad, Hercules, CA, USA) according to the manufacturer’s protocol.

RT-PCR was performed using the SsoAdvanced Universal SYBR Green Supermix (Bio-Rad) on an ABI 3500 RT-PCR Detection System (Applied Biosystems, USA). Table 1 shows the primers that were used to target the QS regulatory gene and *gyrB* (housekeeping gene) (20). The PCR conditions were 95°C for 3 min, followed by 40 cycles of 95°C for 15 seconds, 60°C for 30 seconds, and 72°C for 30 seconds.

**TABLE 1:** Primer sequences (20) and PCR conditions

Gene	Amplicon (bp)	Annealing temp (°C)	Forward primer (5′→3′)	Reverse primer (5′→3′)
*gyrB*	91	60	CAAATGATCACAGCATTTGGTACAG	CGGCATCAGTCATAATGACGAT
*RNAIII*	70	60	TTCACTGTGTCGATAATCCA	TGATTTCAATGGCACAAGAT
*agrA*	79	59	GCACATACACGCTTACAATTGTTG	ACACTGAATTACTGCCACGTTTTAAT
*hla*	99	62	ATGGATAGAAAAGCATCCAAACA	TTTCCAATTTGTTGAAGTCCAAT
*psmα*	179	60	TATCAAAAGCTTAATCGAACAATTC	CCCCTTCAAATAAGATGTTCATATC

### Minimum inhibitory concentration (MIC) and minimum bactericidal concentration (MBC) determination

The MIC and MBC were determined following the guidelines of the Clinical and Laboratory Standards Institute, with slight modifications (21). For MIC, overnight MRSA cultures were adjusted to a 0.5 McFarland standard (≈1 × 10⁸ CFU/mL) and diluted 1:100 in MHB broth to achieve a final inoculum of approximately 1 × 10^5^ CFU/mL. Test compounds (CS, QE, FA, QECS, FACS, and QEFACS) were twofold serially diluted in 96-well microtiter plates to final concentrations ranging from 0.25 to 512 µg/mL. Each well was inoculated with 100 µL of bacterial suspension and incubated statically at 37°C for 18–20 h. MIC was defined as the lowest concentration with no visible bacterial growth compared to the untreated control. For MBC determination, 10 µL aliquots from wells showing no visible growth were plated onto MHA agar and incubated at 37°C for 24 h. The MBC was defined as the lowest concentration of compound resulting in ≥99.9% reduction in CFU compared to the initial inoculum. All assays were performed in at least three independent biological replicates, with each condition tested in triplicate.

### Biofilm inhibition and eradication assays

To evaluate the effects of QE and FA encapsulated in CS on the minimum biofilm inhibitory concentration (MBIC) and minimum biofilm eradication concentration (MBEC) against MRSA, static microtiter plate assays were performed with modifications to previously described protocols (22–24). For the MBIC, overnight MRSA cultures were diluted 1:100 in fresh LB broth and incubated at 37°C for 2 h to reach early exponential phase (25, 26). The bacterial suspension was then further diluted 1:1,000 in LB broth, and 200 µL aliquots were seeded into sterile, flat-bottom 96-well polystyrene microtiter plates. The test compounds were added to the wells at final concentrations of 16, 32, 64, 128, 256, and 512 µg/mL. Control wells containing bacteria treated with 0.05% DMSO served as the negative control and were designated as representing 100% biofilm formation.

The plates were then incubated statically at 37°C for 48 h to allow biofilm formation. After incubation, the culture medium was carefully discarded, and each well was gently washed three times with sterile PBS to remove planktonic and loosely attached cells. The adherent biofilm was subsequently stained with 150 µL of 0.1% (wt/vol) crystal violet solution for 15 min at room temperature. Following staining, the plates were washed thoroughly, air-dried, and the bound crystal violet was solubilized by adding 125 µL of 30% (vol/vol) glacial acetic acid per well. The optical density (OD) was measured at 570 nm using a microplate reader (Startfix-4200, Awareness, USA). The percentage of biofilm inhibition was calculated by comparing the OD values of treated wells with those of the control wells using the following formula:


$$Biofilm\;Inhibition\;(\%)=1-\frac{OD\;of\;treated\;well}{OD\;of\;control\;well}\times 100$$

For MBEC determination, pre-formed MRSA biofilms were generated by incubating bacterial suspensions at 37°C for 48 h. After washing with PBS, fresh medium containing QE or FA (16 µg/mL–512 µg/mL) was added, and plates were incubated for an additional 24 h. The biofilms were then processed for staining and OD measurement as described above for MBIC. MBEC was defined as the lowest concentration capable of eradicating pre-formed biofilms relative to untreated controls. All assays were performed in at least three independent biological replicates, with each condition tested in triplicate.

### Determination of synergistic interactions

The potential synergistic effects between clindamycin and the tested compounds (CS, QE, FA, QECS, FACS, and QEFACS) were evaluated using the checkerboard microdilution method. This approach enables the quantitative assessment of drug interactions through calculation of the fractional inhibitory concentration index (FICI), which integrates the MICs of each agent alone and in combination. The following formulas were used to calculate the fractional inhibitory concentration (FIC) and the FICI:


$$\;\;\;\;\;\;\;{FIC}_{A}=(MIC\;of\;A\;in\;combination)\;/\;(MIC\;of\;\;A\;alone)$$

$$\;\;\;\;\;\;\;{FIC}_{B}=(MIC\;of\;B\;in\;combination)\;/\;(MIC\;of\;B\;\;alone)$$

$$\;\;\;\;\;\;\;FICI={FIC}_{A}+{FIC}_{B}$$

Based on established interpretive criteria, synergistic activity was defined as FICI ≤ 0.5, additive (partial synergy) as 0.5 < FICI ≤ 1.0, indifference as 1.0 < FICI ≤ 4.0, and antagonism as FICI > 4.0 (20, 27). All experiments were conducted in at least three independent biological replicates, with each condition tested in triplicate.

### Hemolysis assay

According to Bernabè et al. (20), with slight modifications, the hemolytic activity of MRSA was assessed by detecting hemoglobin release from red blood cells. MRSA cultures, with or without the test compounds, were cultured at 37°C at a density of 10^6^ CFU/mL. After 16 h, the cultures were centrifuged at 6,000 × *g* at 4°C, and the supernatants were passed through a 0.22 µm filter for sterilization. To prepare each compound, 100 µL was mixed with 900 µL of hemolysin buffer (0.145 M NaCl, 0.02 M CaCl_2_) and 25 µL of defibrinated rabbit blood. The mixtures were then placed on an orbital shaker and incubated at 37°C for 1 h. After incubation, the samples were centrifuged at 8,000 rpm for 1 min at room temperature to pellet intact red blood cells. The supernatants were then collected in a 96-well plate and analyzed at 541 nm. A sterile culture medium served as the 0% hemolysis baseline, while untreated bacterial culture supernatant represented 100% hemolysis. The percentage inhibition of hemolysis was calculated in relation to control cultures. Each experiment was conducted at least twice, with triplicate measurements for each condition.

### Macrophage intracellular killing

Murine macrophage RAW264 cells (Pasteur Institute, Tehran, Iran) were cultured in high-glucose DMEM with 10% FBS and antibiotics at 37°C in 5% CO_2_. MRSA was grown overnight in LB medium, with or without 10 µg of QEFACS. For experiments, RAW264 cells were trypsinized, washed, and resuspended in antibiotic-free DMEM with 1% FBS at 2 × 10⁷ cells/mL, then mixed with MRSA at a 1:1 multiplicity of infection. Samples were centrifuged and incubated to encourage phagocytosis, followed by lysostaphin treatment to remove extracellular bacteria. Phagocytosed bacteria were counted after lysing macrophages and plating on LB agar, with parallel samples incubated 4 extra hours before lysis and counting.

### Cytotoxicity and apoptosis assay

To evaluate cytotoxicity, Vero, L929, and MCF7 cells were plated in 96-well plates at a density that reached 70% confluency and left to incubate overnight to allow attachment. The following day, the cells were treated with different concentrations of the compounds and incubated for 24 h. Cytotoxicity was evaluated using the MTT assay, where 10 µL of MTT solution (5 mg/mL in PBS) was added to each well, followed by incubation for 4 h at 37°C. After incubation, the media was removed, and the formazan crystals formed by metabolically active cells were dissolved in 100 µL of DMSO. The absorbance was measured at 570 nm using a microplate reader, with results normalized to untreated control cells. Data were expressed as percentages of live and dead cells relative to the control group.

To evaluate apoptosis, cells were treated with the compounds for 24 h and then stained with FITC Annexin V Apoptosis Detection Kit (BioLegend, San Diego, CA), according to the manufacturer’s instructions. Annexin V binds to phosphatidylserine on the outer membrane of early apoptotic cells, while propidium iodide (PI) stains the DNA of late apoptotic or necrotic cells. The samples were analyzed by a BD FACSCalibur flow cytometer (Biosciences, San Jose, CA, USA) to determine the percentage of viable (Q4), early apoptosis (Q3), late apoptosis (Q2), and necrosis (Q1) quadrants.

For apoptosis analysis, cells were seeded in six-well plates at a density of 1 × 10^6^ cells per well and allowed to adhere overnight. The following day, cells were treated with individual compounds or combinations at their respective optimized concentrations for 24 h. The treatments included CS alone, FACS, QECS, and QEFACS. Untreated cells served as the control group. Following a 24 h treatment, cells were harvested, washed with PBS, and stained using the FITC Annexin V Apoptosis Detection Kit (BioLegend, San Diego, CA) according to the manufacturer’s instructions. Briefly, cells were resuspended in 100 µL of binding buffer, then incubated with 5 µL of FITC Annexin V and 5 µL of PI for 15 min at room temperature in the dark. After incubation, 400 µL of binding buffer was added to each tube. Samples were analyzed immediately using a BD FACSCalibur flow cytometer (Biosciences, San Jose, CA, USA) with data acquisition set for a minimum of 10,000 events per sample. FITC Annexin V was detected in the FL1-H channel, and PI was detected in the FL2-H channel. For quantitative analysis of apoptosis, gates were set for two regions: M1 (representing viable or low apoptotic cells) and M2 (representing cells with high Annexin V and/or PI staining, indicating apoptosis or necrosis).

### Statistical analysis

All experiments were performed in triplicate, and data are presented as mean ± SD. Statistical analysis was performed using GraphPad Prism 9 software. Differences between treatment groups were analyzed by one-way analysis of variance followed by *post hoc* Tukey’s test. A *P*-value <0.05 was considered statistically significant. The flow cytometry analysis was conducted using FlowJo software (version 10.5.3, Tree Star Incorporated, Ashland, OR).

## RESULTS

### DLS and zeta potential

The average particle sizes for the individual components and combinations are shown in Table 2. CS nanoparticles had an average particle size of 250 nm, QE nanoparticles measured 220 nm, and FA nanoparticles had a size of 230 nm. The combination of CS, QE, and FA resulted in larger particles, averaging 300 nm, suggesting that combining agents leads to aggregation or increased particle size. The polydispersity index (PDI) values indicated good uniformity of particle distribution (PDI <0.3 for all formulations). The surface charge (zeta potential) of the nanoparticles was measured to assess their stability in suspension. As shown in Table 2, CS nanoparticles exhibited a highly positive zeta potential of +45 mV, contributing to their stability in aqueous media. QE and FA nanoparticles had zeta potentials of −20 mV and −18 mV, respectively. The QEFACS nanoparticles exhibited a zeta potential of +35 mV, indicating a reduction in surface charge due to the interaction between positively charged CS and negatively charged QE and FA (Table 2).

**TABLE 2:** Summary of DLS, zeta potential, and encapsulation efficiency data^*a*^

Treatment	Particle size (nm)	PDI	Zeta potential (mV)	Encapsulation efficiency (%)
CS	250 ± 10	0.25	+45 ± 3	NA
QE	220 ± 8	0.22	−20 ± 2	NA
FA	230 ± 9	0.24	−18 ± 3	NA
QECS	275 ± 12	0.26	+30 ± 3	82 ± 2
FACS	280 ± 11	0.28	+32 ± 2	78 ± 3
QEFACS	300 ± 15	0.30	+35 ± 4	75 ± 3

^*a*^ Polydispersity index (PDI). CS; Chitosan, QE; Quercetin, FA; Ferulic Acid, QECS; encapsulation of QE and CS, FACS; encapsulation of FA and CS, QEFACS; encapsulation of QE, FA, and CS; NA, not applicable.

### Encapsulation efficiency

The encapsulation efficiency (EE%) of QE and FA in the CS nanoparticles was calculated based on UV-Vis spectrophotometric analysis of the unencapsulated compounds. The encapsulation efficiency was high for both compounds, with QE showing an encapsulation efficiency of 82% and FA 78%. When combined, the encapsulation efficiency of the QEFACS was slightly reduced to 75%, likely due to competition for binding sites in the CS matrix (Table 2).

### TGA and XRD analysis

**Fig 1:**
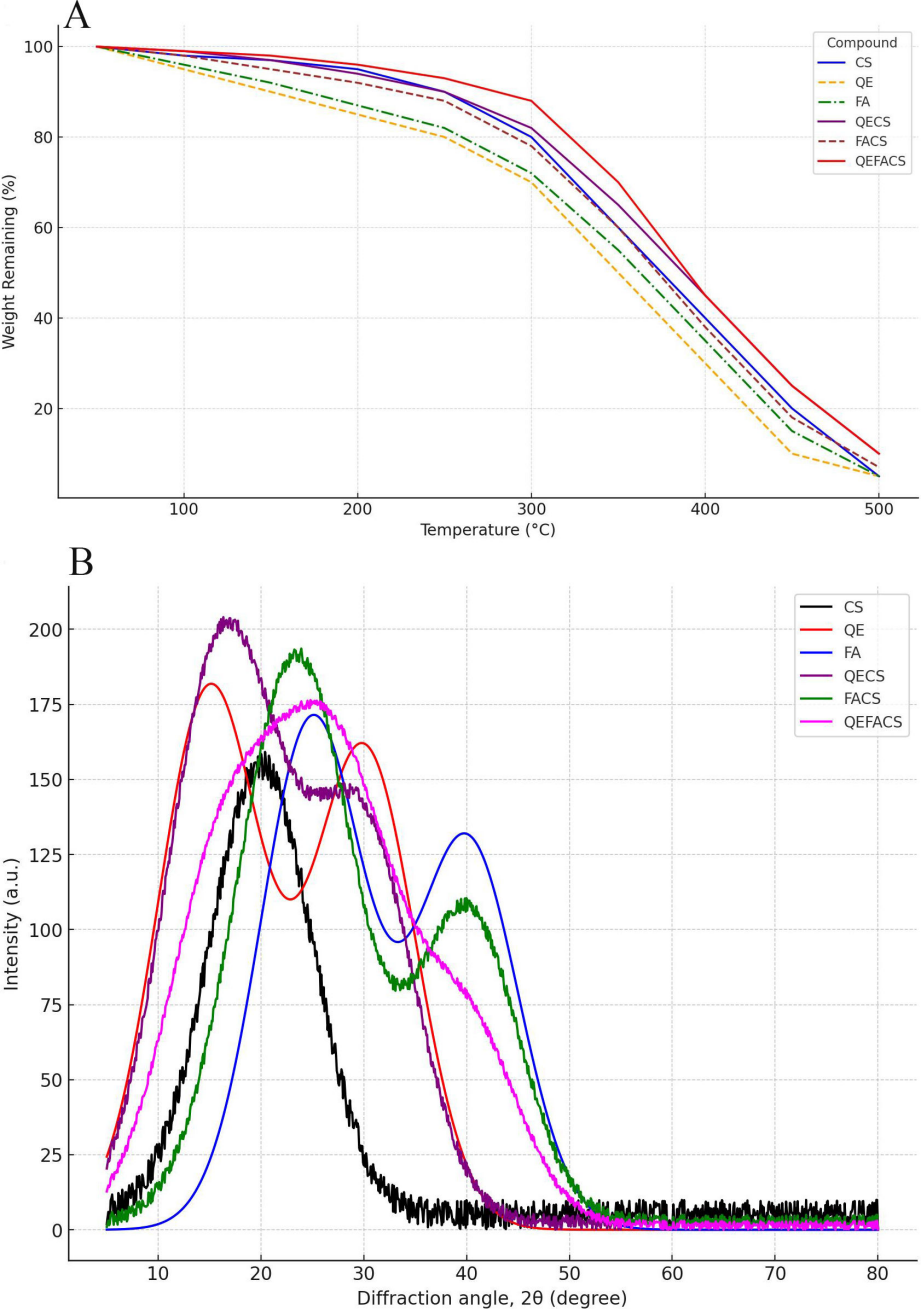
(**A**) TGA analysis of compounds. In the TGA plot, single compounds like FA and QE degrade faster than CS due to their simpler, more reactive structures. The QECS and FACS display intermediate stability, benefiting partially from chitosan’s protective matrix. The QEFACS shows the highest thermal stability, likely due to the combined protective effects of chitosan encapsulating both quercetin and ferulic acid. (**B**) XRD plot for the compounds and their combinations. CS shows a broad peak around 20°, indicating semi-crystallinity. QE and FA display sharp peaks, characteristic of crystalline structures. QECS, FACS, and QEFACS combinations show reduced peak intensities, indicating partial amorphization, especially in QEFACS, suggesting successful encapsulation in the CS matrix.

The TGA data demonstrated that pure CS nanoparticles remained stable up to approximately 300°C, with significant weight loss observed only after this temperature, attributed to polymer degradation. In contrast, free QE and FA began to show weight loss around 150°C, likely due to moisture evaporation and the decomposition of their organic structures. Encapsulated forms QECS and FACS exhibited improved thermal stability, with decomposition beginning closer to 200°C, while QEFACS displayed the highest stability among all samples (Fig. 1A), with degradation initiated at approximately 270°C. This indicates that the chitosan matrix contributes to enhanced thermal resistance, likely by creating a protective barrier around the organic components, thereby delaying their decomposition.

The XRD analysis provided insight into the crystalline structure of each sample. Pure CS displayed a broad peak around 2θ = 20°, indicative of its semi-crystalline nature. Free QE and FA exhibited sharp peaks at specific 2θ angles, reflecting their crystalline forms. However, the encapsulated forms QECS, FACS, and QEFACS demonstrated a noticeable reduction in peak intensity, especially in the QEFACS sample (Fig. 1B). This reduction in crystallinity suggests partial amorphization of QE and FA within the CS matrix, implying successful encapsulation that disrupts the crystalline structure of the compounds. This transformation to a more amorphous state is advantageous, as it could improve solubility and bioavailability, enhancing the functional properties of QE and FA when delivered in encapsulated nanoparticle form. Together, these TGA and XRD results underscore the role of chitosan encapsulation in enhancing the stability and modifying the structural characteristics of QE and FA, supporting the potential of QEFACS as a robust, bioavailable formulation for biomedical applications.

### SEM analysis

**Fig 2:**
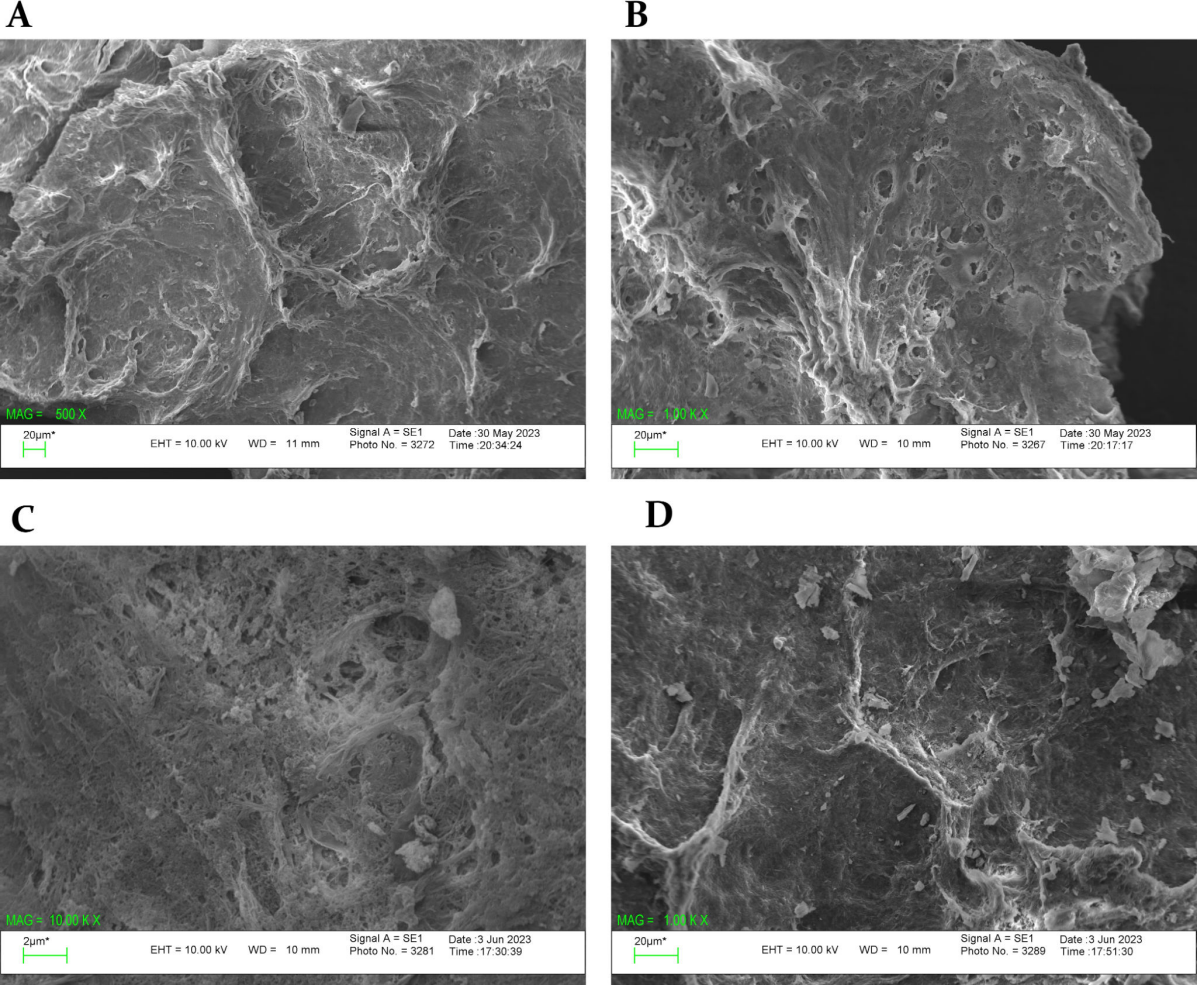
SEM images of CS, QECS, FACS, and QEFACS nanoparticles. Panel (**A**) shows the surface morphology of pure CS, with a relatively smooth and dense structure. Panel (**B**) reveals increased surface roughness and porosity due to the encapsulation of FA into the CS matrix. Panel (**C**) displays a more irregular and porous surface morphology, indicating successful encapsulation of QE. Panel (**D**) exhibits the most complex morphology, with a highly rough and porous surface, reflecting the combined incorporation of both FA and QE in the CS matrix. Scale bars represent 20 µm for images A, B, and D, and 2 µm for image C.

The SEM images of CS, QECS, FACS, and QEFACS nanoparticles reveal varied, non-spherical morphologies with distinct surface textures for each formulation. Pure CS nanoparticles exhibit a relatively smooth, irregular shape, indicative of the polysaccharide’s natural structure. Encapsulation of quercetin in QECS results in rougher surfaces and increased irregularity, likely due to QE’s interaction with the CS matrix, disrupting uniform particle formation. FACS nanoparticles, which incorporate FA, display even more pronounced surface roughness and noticeable aggregation, suggesting stronger interactions between FA and chitosan. This clumping effect is further amplified in QEFACS, where the combination of both QE and FA within the CS matrix produces highly irregular, textured particles with significant aggregation (Fig. 2). These morphological changes, particularly in QEFACS, indicate successful encapsulation and potentially enhanced functional properties, as the increased surface roughness and expanded surface area could improve the nanoparticles’ interactions in bioactive applications.

### Determination of synergistic interactions

The checkerboard microdilution assay revealed that the combination of clindamycin with selected chitosan-encapsulated phytochemicals resulted in enhanced antimicrobial activity against MRSA. Notably, the co-administration of QEFACS with clindamycin demonstrated strong synergistic effects, with FICI values ≤0.5, indicating a significant potentiation of clindamycin activity. The combination of QECS or FACS with clindamycin produced additive interactions (0.5 < FICI ≤ 1.0), while unencapsulated QE and FA exhibited mainly indifferent effects when combined with clindamycin (1.0 < FICI ≤ 4.0). Importantly, no antagonistic interactions (FICI > 4.0) were observed for any of the tested formulations (Table 3).

**TABLE 3:** Antimicrobial and antibiofilm activities of compound–clindamycin combinations against MRSA^*a*^

Compound/treatment	MIC (µg/mL, alone)	MIC (µg/mL, combination)	MBC (µg/mL)	MBIC (µg/mL)	MBEC (µg/mL)	FICI	Interpretation	MRSA inhibition (%) ± SD	Biofilm reduction (%) ± SD
CS + clindamycin	128	64 + 8	128	64	128	1.0	Indifferent	30 ± 3	35 ± 4
QE + clindamycin	64	32 + 8	64	32	64	1.0	Additive	45 ± 4	40 ± 3
FA + clindamycin	64	128 + 32	256	128	256	4.0	Indifferent	20 ± 3	25 ± 3
QECS + clindamycin	32	16 + 8	32	16	32	1.0	Additive	60 ± 4	60 ± 5
FACS + clindamycin	32	32 + 16	64	64	128	2	Indifferent	50 ± 4	55 ± 4
QEFACS + clindamycin	16	4 + 4	8	4	8	0.5	Synergy	95 ± 2	92 ± 3
Clindamycin alone	16	NA	64	16	64	NA	Indifferent	40 ± 3	60 ± 4

^*a*^ Interpretation criteria: synergy: FICI ≤ 0.5; additive: 0.5 < FICI ≤ 1.0; indifferent: 1.0 < FICI ≤ 4.0; antagonistic: FICI > 4.0. Data are presented as mean ± SD of three independent experiments; NA, not applicable.

### Biofilm reduction and MRSA growth inhibition

For MRSA growth inhibition, individual treatments with CS, QE, and FA achieved moderate inhibition levels, reaching 30%, 45%, and 40%, respectively (Table 3). The encapsulated forms demonstrated greater efficacy, with QECS and FACS showing 60% and 55% inhibition, while the QEFACS combination achieved the highest inhibition at 85% (*P* < 0.01). The combination of QEFACS with clindamycin demonstrated the lowest MIC and the highest FIC index, indicating strong synergy (*P* < 0.001).

In terms of biofilm reduction, CS, QE, and FA alone reduced biofilm formation by 35%, 40%, and 25%, respectively. The encapsulated combinations displayed superior results, with QECS and FACS reaching 60% and 55% biofilm reduction. Notably, QEFACS exhibited the most pronounced effect, reducing biofilm formation by 92% (*P* < 0.001), suggesting enhanced potency in biofilm inhibition when both active compounds are co-encapsulated within the CS matrix.

**Fig 3:**
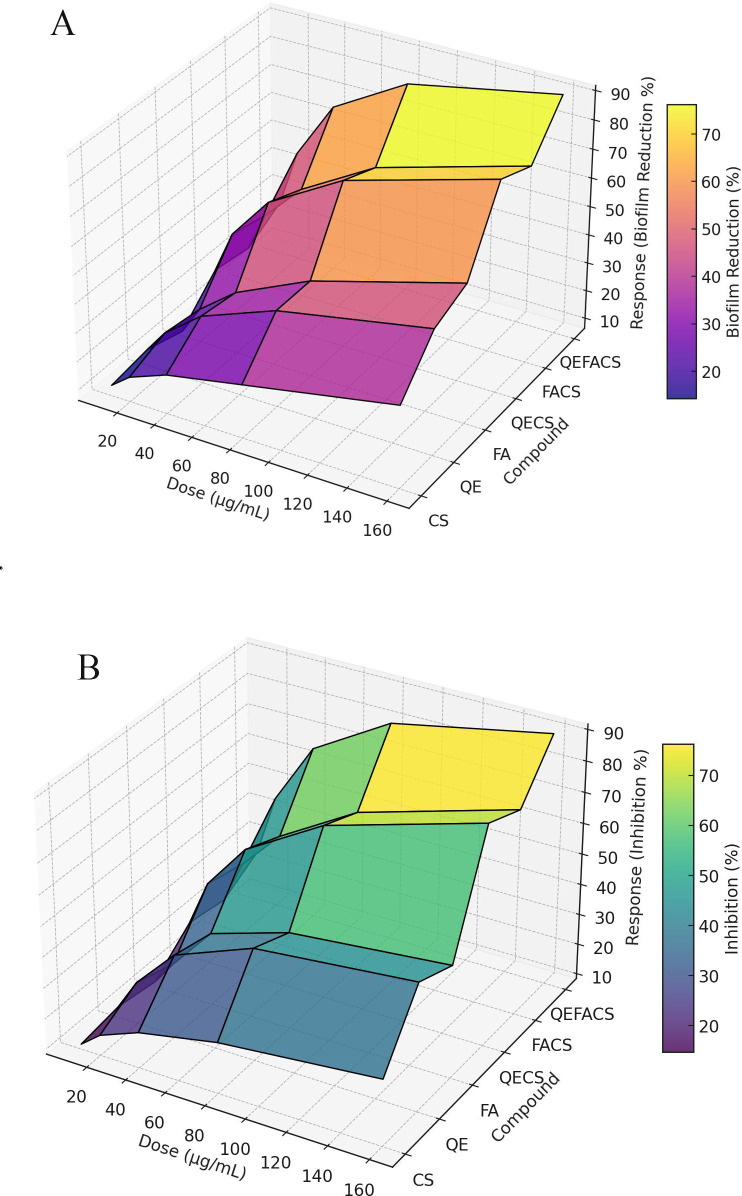
3D surface plot for growth inhibition and MRSA growth inhibition. The (**A**) plot represents biofilm reduction in response between increasing doses (10 to 160 µg/mL), showing a marked reduction in biofilm formation, particularly for the encapsulated compounds (QECS, FACS, QEFACS). The (**B**) plot illustrates the relationship to the same dosage range and bacterial inhibition for each compound (CS, QE, FA, QECS, FACS, QEFACS), with QEFACS demonstrating the highest inhibition across doses.

The 3D surface plots demonstrate a clear dose-dependent effect of various compounds (CS, QE, FA, QECS, FACS, and QEFACS) on both inhibition of MRSA growth (Fig. 3A) and biofilm reduction (Fig. 3B). For bacterial inhibition, all compounds show increased efficacy with rising doses, with the encapsulated formulations (especially QEFACS) achieving the highest levels of inhibition at maximum doses. Similarly, biofilm reduction shows a positive correlation with dosage, with QEFACS and other encapsulated forms (QECS and FACS) producing the most substantial reductions, suggesting enhanced efficacy when QE and FA are encapsulated with CS. Notably, QEFACS achieves the most substantial reduction, reaching up to 90% biofilm reduction at higher doses (160 µg/mL).

### Effects on QS gene expression in MRSA

**Fig 4:**
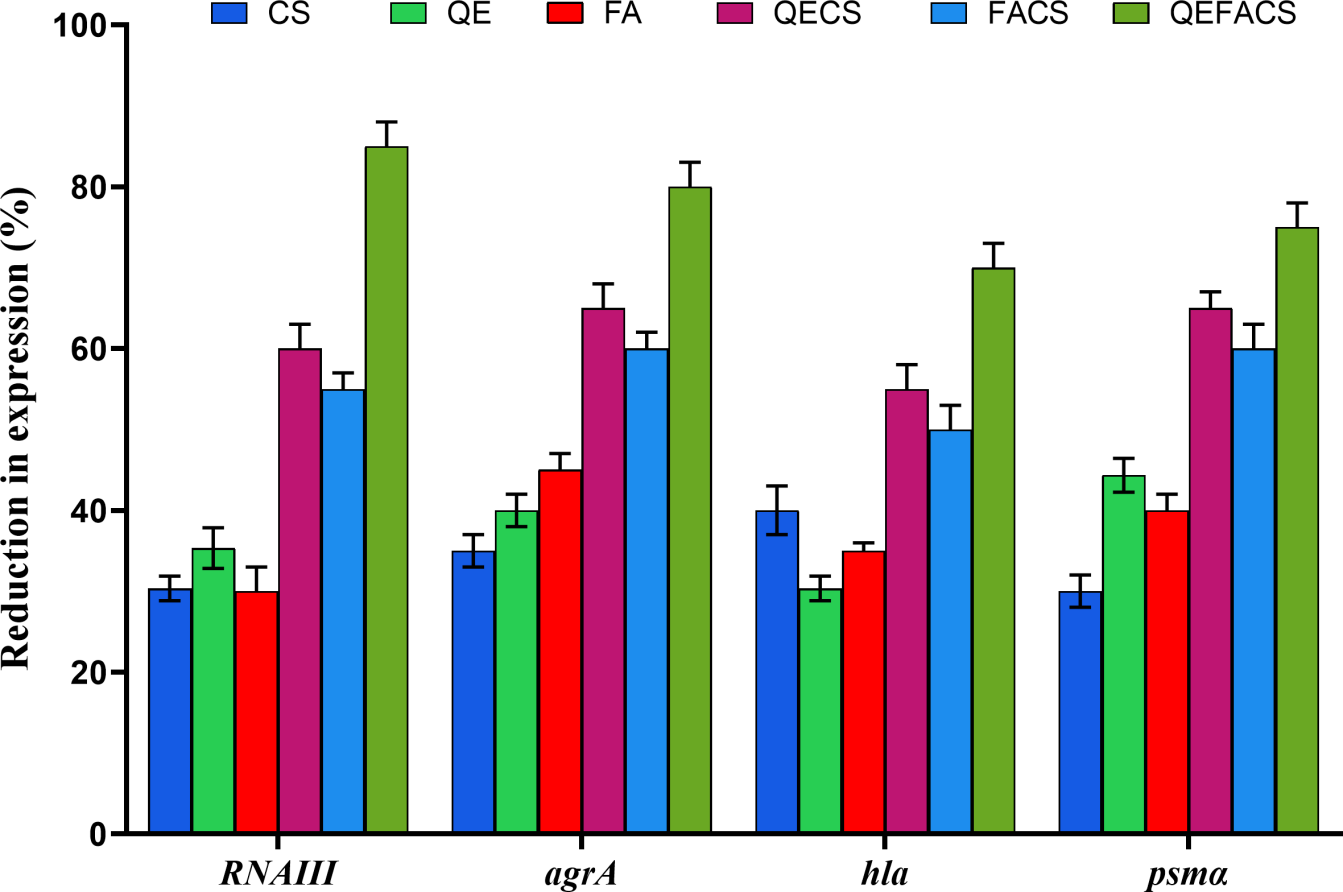
Effects of compounds on QS gene expression in MRSA. Reduction in expression of *RNAIII*, *agrA*, *hla*, and *psmα* genes following different treatment conditions. The bar graph represents the percentage reduction in gene expression relative to the untreated control. Data are shown as mean ± SD from three independent experiments.

All treatments significantly reduced the expression of these QS-related genes compared to the untreated control, with combination therapies demonstrating the greatest reduction. Specifically, CS alone reduced *RNAIII*, *agrA*, *hla*, and *psmα* expression by 30%–40%, while QE and FA individually caused reductions in the range of 30%–45%. Notably, QEFACS demonstrated significant downregulation, with *RNAIII*, *agrA*, *hla*, and *psmα* expression reduced by 85%, 80%, 70%, and 75%, respectively. Synergistic effects were particularly evident when QE or FA was combined with CS, significantly enhancing QS inhibition (*P* < 0.001) compared to the use of individual agents, especially for *RNAIII* and *psmα* (Fig. 4).

### Hemolysis assay

The hemolysis assay results demonstrate that each tested compound significantly inhibits MRSA-induced hemolysis, with varying levels of effectiveness. CS alone achieved 35% inhibition of hemolysis (*P* < 0.05), while QE and QECS complex exhibited higher inhibition levels of 55% and 60%, respectively, both statistically significant at *P* < 0.01. The FA provided 75% inhibition, while the FACS complex inhibited hemolysis by 65%, both showing high statistical significance with *P* < 0.001. The QEFACS complex demonstrated the highest inhibition of hemolysis at 85%, with a *P*-value of <0.001. These results confirm that all compounds reduce hemolysis significantly compared to the untreated control, with QEFACS, FA, and FACS displaying the highest levels of inhibition (Table 4).

**TABLE 4:** Inhibition of hemolysis

Compound^*a*^	Average absorbance at 541 nm	% Hemolysis (compared to control)	% Inhibition of hemolysis	*P*-value (compared to untreated control)
CS	0.67	65%	35%	*P* < 0.05
QE	0.45	45%	55%	*P* < 0.01
FA	0.25	25%	75%	*P* < 0.001
QECS	0.40	40%	60%	*P* < 0.01
FACS	0.35	35%	65%	*P* < 0.001
QEFACS	0.15	15%	85%	*P* < 0.001

^*a*^ CS; Chitosan, QE; Quercetin, FA; Ferulic Acid, QECS; encapsulation of QE and CS, FACS; encapsulation of FA and CS, QEFACS; encapsulation of QE, FA, and CS.

### Macrophage intracellular killing

The intracellular killing results show progressive reductions in MRSA CFUs across both 1 h and 4 h incubation points, with each compound enhancing macrophage-mediated bacterial killing to different extents. CS alone demonstrated minimal early reduction (10% at 1 h, *P* < 0.05), resulting in a total 20% reduction after 5 h, indicating moderate macrophage support. QE produced a greater initial reduction of 25% at 1 h and 40% by 5 h (*P* < 0.01), suggesting improved bacterial clearance. FA showed a significant 30% reduction at 1 h and reached 50% reduction at 5 h (*P* < 0.001), making it more effective than QE alone. Combinations of compounds further enhanced killing; QECS achieved 32% reduction at 1 h and 55% at 5 h (*P* < 0.01), and FACS resulted in 35% reduction at 1 h and 60% at 5 h (*P* < 0.001). The most effective compound, QEFACS, reduced MRSA by 45% within 1 h and reached 75% reduction at 5 h (*P* < 0.001), demonstrating the highest level of bacterial clearance (Table 5).

**TABLE 5:** Macrophage intracellular killing

Compound^*a*^	Initial CFU count	CFU count after 1 h incubation	CFU count after 5 h incubation	% Reduction after 5 h	*P*-value (compared to untreated control)
CS	1.0 × 10⁵	9.0 × 10⁴	8.0 × 10⁴	20%	*P* < 0.05
QE	1.0 × 10⁵	7.5 × 10⁴	6.0 × 10⁴	40%	*P* < 0.01
FA	1.0 × 10⁵	7.0 × 10⁴	5.0 × 10⁴	50%	*P* < 0.001
QECS	1.0 × 10⁵	6.8 × 10⁴	4.5 × 10⁴	55%	*P* < 0.01
FACS	1.0 × 10⁵	6.5 × 10⁴	4.0 × 10⁴	60%	*P* < 0.001
QEFACS	1.0 × 10⁵	5.5 × 10⁴	2.5 × 10⁴	75%	*P* < 0.001

^*a*^ CS; Chitosan, QE; Quercetin, FA; Ferulic Acid, QECS; encapsulation of QE and CS, FACS; encapsulation of FA and CS, QEFACS; encapsulation of QE, FA, and CS.

### Cytotoxicity and apoptosis assay

**Fig 5:**
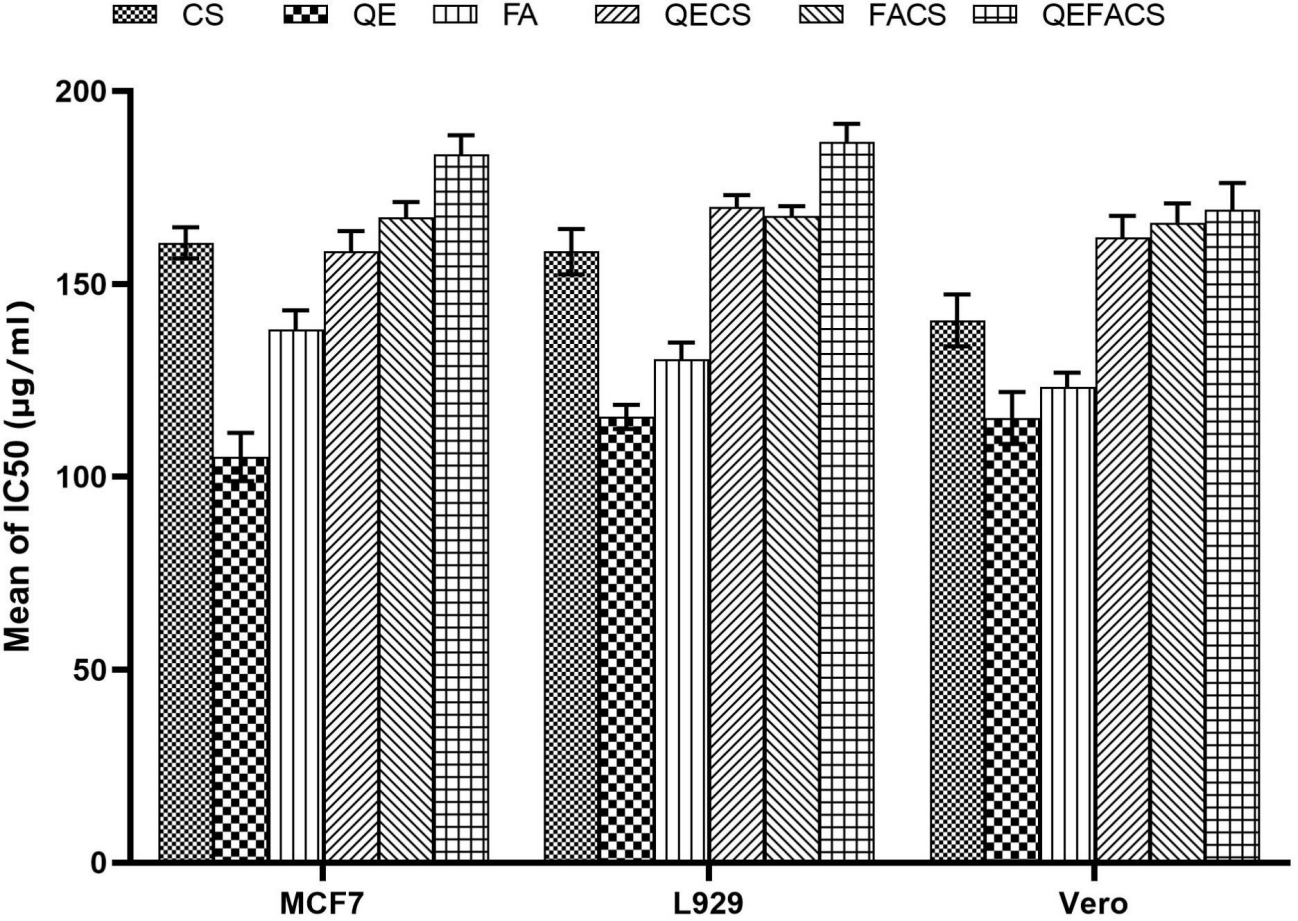
Cytotoxicity of compounds CS, QE, FA, QECS, FACS, and QEFACS against MCF7, L929, and Vero cell lines. The mean IC_50_ values (µg/mL) were determined after 24 h of treatment and are presented as bar graphs with error bars indicating the standard deviation.

The cytotoxicity data reveal that all the compounds (CS, QE, FA, QECS, FACS, and QEFACS) exhibit dose-dependent cytotoxic effects across all three cell lines (Vero, L929, and MCF7). As the concentration of each compound increased, cell viability significantly decreased. Among the compounds, QE and FA showed the most pronounced cytotoxicity, particularly at higher concentrations, resulting in as low as 10% viability in MCF7 cells. Complex formulations like QECS, FACS, and QEFACS demonstrated slightly lower cytotoxicity compared to the free compounds (QE and FA), particularly in MCF7 and L929 cells, suggesting a potential reduction in toxicity when combined with CS (Fig. 5). The CS compound alone also exhibited dose-dependent toxicity, though it was less toxic than the free forms of QE and FA. Overall, while all compounds showed reduced cell viability at higher concentrations, they did not reach critical toxicity thresholds at lower concentrations (≤ 100 µg/mL), highlighting their potential for therapeutic applications.

**Fig 6:**
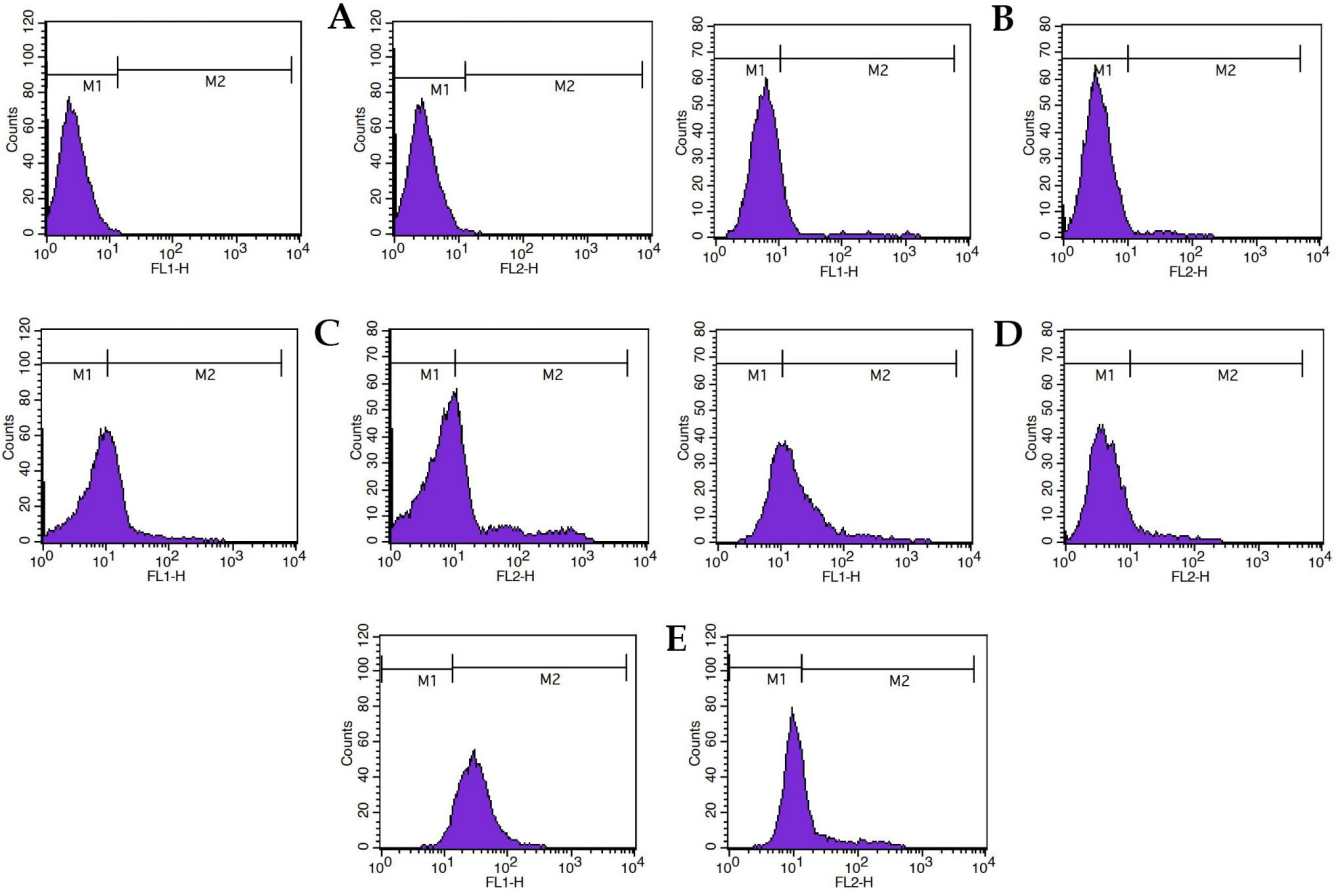
Flow cytometry analysis of apoptotic induction in cells treated with various compounds. Histograms display fluorescence intensity in the FL1-H and FL2-H channels, with cells distributed across M1 (low fluorescence, indicating viable cells) and M2 (higher fluorescence, indicating apoptotic or necrotic cells) regions. Panel (**A**) represents the control group without apoptosis, showing primarily viable cells in the M1 region. Panels (**B**) to (**E**) display increasing apoptosis levels: (**B**) CS alone induces 15% apoptosis, (**C**) FACS induces 40% apoptosis, (**D**) QECS induces 65% apoptosis, and (**E**) QEFACS induces the highest apoptosis at 82%.

The flow cytometry analysis compares apoptotic induction across treatments (CS, FACS, QECS, and QEFACS) with an untreated control, focusing on shifts toward the M2 region. In the control, most cells are in the M1 region, indicating minimal apoptosis. CS alone induces a modest apoptosis rate of 15%, while FACS significantly increases apoptosis to 40%, showing enhanced effectiveness over CS alone. QECS further boosts apoptosis to 65%, suggesting a synergistic effect with the addition of QE. The QEFACS treatment shows the strongest apoptotic response, with 82% of cells in M2, highlighting the potent synergy of QE, FA, and CS in inducing apoptosis (Fig. 6).

## DISCUSSION

We evaluated herein the inhibitory effect of CS, QE, and FA, alone and in combination, on growth, biofilm formation, and QS gene expression of MRSA and their synergistic interaction with clindamycin, which enhances the action of the antibiotic against MRSA. The results showed that these compounds, especially in combination, highly inhibit the growth of MRSA, the formation of its biofilms, and the transcription of major QS system genes such as *RNAIII*, *agrA*, *hla*, and *psmα*.

QE, FA, and CS inhibited the growth of MRSA by 40%, 45%, and 38%, respectively. However, in combination (QEFACS), MRSA growth was inhibited by 85%, showing a strong synergistic effect. This enhanced antibacterial action most likely results because these compounds have complementary mechanisms that disrupt bacterial membranes, inhibiting critical cellular processes and a reduction in the production of virulence factors. Our observations have been confirmed by previous studies that reported that a combination of CS with polyphenols, such as QE, exerts synergistic antibacterial activity (28, 29). In this study, the membrane-disrupting action of CS was combined with QE and FA-mediated inhibition of bacterial cellular functions, therefore providing an effective synergistic effect on MRSA growth inhibition.

Our results also demonstrate that CS, QE, and FA can reduce the biofilm formation in MRSA. Biofilms are one of the factors that complicate infection treatment, as they protect bacteria from both antibiotics and the host immune system, representing a major challenge for healthcare professionals (30). To objectively substantiate the observed synergistic and additive effects, FICI values for all combinations were determined (Table 3). Notably, the QEFACS–clindamycin combination yielded a FICI of 0.5, meeting the widely accepted threshold for synergy (FICI ≤ 0.5) and corresponding to a 95% reduction in MRSA growth and a 92% reduction in biofilm formation. The other combinations yielded different outcomes: CS + clindamycin, FA + clindamycin, and FACS + clindamycin exhibited “indifferent” effects (FICI = 1–4), whereas QE + clindamycin and QECS + clindamycin showed “additive” effects (FICI = 1.0). Reporting these FICI data provides an objective and quantitative framework for interpreting pharmacodynamic interactions, reinforcing the evidence that QEFACS combined with clindamycin exerts true synergistic effects rather than merely additive inhibition. The ability of CS to inhibit biofilm formation has been widely reported in the literature (31–33). Different studies have shown that CS exerts an inhibitory action on biofilms, as it interacts with the extracellular polymeric substances (EPS) that hold biofilms together and exerts its action on bacterial adhesion-aggregation (34, 35). Our results are in line with previous studies, such as the report by Li et al. (35), that demonstrated that CS could significantly reduce biofilm formation in a variety of bacterial strains (35).

QE and FA also seem to play a significant role in biofilm inhibition, probably as a result of the disruption of QS signaling and downregulation of genes associated with biofilm (36, 37). A study by Ouyang et al. (38) demonstrated that QE inhibited biofilm formation in *Pseudomonas aeruginosa* by disrupting QS signaling. Similarly, FA has been shown to inhibit biofilm formation in *Escherichia coli* and *Staphylococcus epidermidis* through interference with QS and the downregulation of biofilm-associated genes (39).

The significant biofilm reduction observed with the QEFACS in our study highlights the potential of these compounds to be used as alternative or adjunct therapies to combat biofilm-associated infections, particularly in the context of antibiotic resistance. A combination targeting various aspects of biofilm formation, such as bacterial adherence, QS regulation, and EPS integrity by QEFACS, may offer a potent approach against the treatment of difficult biofilm-associated infections.

One of the most interesting findings in this research is the synergy between CS, QE, FA, and clindamycin. The combination of such compounds with clindamycin enhanced the effectiveness of this antibiotic against MRSA and decreased its MIC by more than 50%. More studies are now looking at the synergistic effects of natural compounds with antibiotics to make antibiotics more effective against resistant bacteria (40). In particular, CS increased the activity of various antibiotics, including clindamycin, due to the increased membrane permeability and hence facilitating the entry of antibiotics into bacterial cells. In particular, CS increased the activity of antibiotics by increasing bacterial membrane permeability, hence allowing the greater entry of the antibiotic into the cells (41). Previous studies have indicated that CS could reduce the MIC of antibiotics such as vancomycin and gentamicin in combination treatments (42, 43).

QE and FA have also been shown to exhibit synergistic effects with antibiotics. For example, QE has been reported to enhance the activity of antibiotics such as ciprofloxacin and tetracycline against resistant strains of *Pseudomonas aeruginosa* (44).

The enhanced antibacterial activity of clindamycin in combination with QEFACS observed in our study is likely due to the disruption of bacterial membranes, coupled with interference in essential cellular processes, thereby rendering MRSA more susceptible to antibiotic treatment.

Another important finding is the significant reduction in QS gene expression in MRSA following treatment with QEFACS. These compounds substantially downregulated key QS-regulated genes, including *RNAIII*, *agrA*, *hla*, and *psmα*, with the combination treatment showing the most potent inhibitory effect. In *S. aureus*, the QS system regulates virulence factor production, biofilm formation, and toxin expression (9). Disrupting QS signaling can reduce pathogenicity without directly killing bacteria, which might help minimize resistance development. Our results align with previous research demonstrating that CS, QE, and FA can inhibit QS signaling. For instance, studies have shown that CS reduces the expression of virulence genes like *agrA* and *hla* in MRSA, leading to decreased hemolysin production and virulence (45). QE has been reported to inhibit QS in *Pseudomonas aeruginosa* by downregulating the expression of QS-regulated genes, including those involved in virulence factor production (46). Downregulation of *RNAIII*, *agrA*, *hla*, and *psmα* in our study hints that CS, QE, and FA work synergistically to effectively weaken the virulence of MRSA through QS interference. This is an important finding because it underlines the potential of these compounds to be used as anti-virulence agents, decreasing the pathogenicity of MRSA without fully depending on bacterial killing, hence opening another way besides traditional antibiotic treatments.

### Conclusion

The combination of CS with QE and FA exerts strong inhibitory effects on MRSA growth, biofilm formation, and QS gene expression. The clear synergy seen with clindamycin suggests good potential for these natural compounds to enhance antibiotic effectiveness against resistant bacterial strains. These results are an indication that CS, QE, and FA, either in single doses or in combination, are good candidates for the production of new drugs to combat MRSA and other antibiotic-resistant pathogens. Translational studies, as well as clinical trials, are necessary to verify these observations and to prove their therapeutic potential in full.

## Data Availability

The authors confirm that the data supporting the findings of this study are available within the article materials.
